# Lectures on the Diagnosis and Treatment of Diseases of the Lungs

**Published:** 1841-06-19

**Authors:** W. W. Gerhard


### MEDICAL EXAMINER.

DEVOTED TO MEDICINE, SURGERY, AND THE COLLATERAL SCIENCES.

No. 25.] PHILADELPHIA, SATURDAY, JUNE 19, 1841. [Vol. IV.

### LECTURES ON THE DIAGNOSIS AND TREATMENT OF DISEASES OF THE LUNGS.

BY W. W. GERHARD, M. D.

### LECTURE XIII.—(*Continued.*)

#### TUBERCLES OF THE BRONCHIAL GLANDS.

The bronchial glands are, at the early periods of life, more subject to tuberculous deposit than the lungs themselves. This tendency to tubercle exists in the bronchial glands to a much higher degree than in any other of the lymphatic ganglia. It is highly developed in children, but gradually declines as individuals advance in life, and in old age the bronchial glands are scarcely ever affected, except as a consequence of previous disease of the lungs. The relative frequency of tubercles in the bronchial glands of children compared with the lungs, is not less than five to four ; this is of course reversed after the age of puberty.

The development of tubercles in the bronchial glands occurs nearly as in other solid structures of the body ; scattered points of tuberculous substance are gradually deposited in the structure of the glands, surrounded by the original tissue, which remains for a considerable time nearly in the healthy state ; sometimes, however, it is swollen and more vascular than usual, but more frequently it is quite pale, and infiltrated with the gelatinous substance which is in many cases the early stage of tuberculous matter. As the quantity of tubercle increases, that of glandular structure gradually becomes less, until the whole tissue of the gland is absorbed, and is replaced by tubercle. It is then much larger than the original gland, and the capsule which encloses it gradually thickens during the process of softening. After softening has followed, adhesion occurs between the glands and the adjacent large bronchial tube, until the contained matter is evacuated by an ulcerated opening. In most instances, however, no softening occurs, but the tuberculous matter becomes hard and dry, and is converted into a calcareous matter, surrounded by the capsule. This substance often becomes extremely hard and solid, and generally remains in this state during life. The tuberculous disease of the bronchial glands is, therefore, much less unfavourable than that of the lungs, and is essentially curable.

The symptoms of tubercles in the bronchial glands are extremely obscure. Indeed they cannot, in the large majority of cases, be recognized except by the signs of a general scrofulous diathesis. As this rarely occurs in children without a deposit of tubercle in the bronchial glands, we may safely infer that the local disease exists, if we discover the symptoms of the general disorder. In such cases no possible disadvantage results from the difficulty of diagnosis, for the disease is without influence upon the lungs. In other cases the tuberculous glands attain a considerable size, and press upon the trachea, obstructing the respiration, and irritating the bronchial mucous membrane. The symptoms of catarrh, however, differ a little from those of ordinary bronchitis; the cough is frequent, but occurs in paroxysms, very much resembling, in many cases, the fits of whooping cough, and, on auscultation, it is found that the respiration is extremely feeble in one or both lungs, while the percussion is quite sonorous. The feebleness of respiration is the only permanent sign, and depends upon the contraction of the larger tubes from the pressure upon them. The expirations are at times wheezing, and, as it were, protracted, but not permanently so. As these are the only symptoms of tubercles of the bronchial glands, and are by no means limited to this lesion, the diagnosis depends at last upon the comparison of these comparatively unimportant local signs with the general indications of a tuberculous diathesis.

The treatment of tubercles of the bronchial glands consists entirely in those means which tend to counteract the scrofulous or tuberculous diathesis, and chiefly in the use of iodine and vegetable alteratives. The use of these remedies should be continued for a long period, if the stomach of the individual be not irritated by their employment; if it should be, they must be immediately discontinued, and, after a time, renewed; free exercise in the open air, and a healthy invigorating diet, are necessary adjuvants in the treatment.

